# Molecular engineering of antibodies for site-specific covalent conjugation using CRISPR/Cas9

**DOI:** 10.1038/s41598-018-19784-2

**Published:** 2018-01-29

**Authors:** Makan Khoshnejad, Jacob S. Brenner, William Motley, Hamideh Parhiz, Colin F. Greineder, Carlos H. Villa, Oscar A. Marcos-Contreras, Andrew Tsourkas, Vladimir R. Muzykantov

**Affiliations:** 1 Department of Pharmacology, The Perelman School of Medicine, University of Pennsylvania, Philadelphia, PA USA; 2 Department of Neurology, Johns Hopkins University, Baltimore, MD USA; 3 Department of Bioengineering, University of Pennsylvania, Philadelphia, PA USA

## Abstract

Site-specific modification of antibodies has become a critical aspect in the development of next-generation immunoconjugates meeting criteria of clinically acceptable homogeneity, reproducibility, efficacy, ease of manufacturability, and cost-effectiveness. Using CRISPR/Cas9 genomic editing, we developed a simple and novel approach to produce site-specifically modified antibodies. A sortase tag was genetically incorporated into the C-terminal end of the third immunoglobulin heavy chain constant region (CH3) within a hybridoma cell line to manufacture antibodies capable of site-specific conjugation. This enabled an effective enzymatic site-controlled conjugation of fluorescent and radioactive cargoes to a genetically tagged mAb without impairment of antigen binding activity. After injection in mice, these immunoconjugates showed almost doubled specific targeting in the lung vs. chemically conjugated maternal mAb, and concomitant reduction in uptake in the liver and spleen. The approach outlined in this work provides a facile method for the development of more homogeneous, reproducible, effective, and scalable antibody conjugates for use as therapeutic and diagnostic tools.

## Introduction

Monoclonal antibody (mAb)-based therapeutics have emerged as very effective tools for treatment of a wide-spectrum of diseases and are already mainstays in the treatment of cancer and autoimmune diseases. Burgeoning subfields of immunotherapeutics include mAb conjugates with drugs, probes and nanoparticles that have gained considerable attention for their clinical utility. Despite the huge potential of antibody-drug conjugates, there have been many challenges in the field which have limited its growth and hindered regulatory success. These challenges comprise of limited control over the number of drug molecules conjugated, reduced affinity and stability of modified antibodies, and limitations to the number of drugs or combinations of drugs conjugated^1–6^. Molecular engineering of antibodies with genetically encoded sequences primed for site-specific conjugation could resolve these issues. Traditionally, genetic modification of mAbs required the sequencing of the hybridoma antibody variable regions, and subsequent cloning into production cell lines where sequence modifications are made, which can be challenging, time-consuming, and often prohibitively expensive for early-stage and academic therapeutic discovery projects.

CRISPR/Cas9 RNA-guided DNA nucleases programmed to site-specifically modify targeted sequences in the genome could revolutionizing biomedicine^7–9^. In particular, the CRISPR technology has the potential to advance the fields of antibody engineering. CRISPR/Cas9 was recently applied for modification of mouse and human immunoglobulin genes to induce class-switch recombination and generate different IgH subclasses^10^. The technology has been also used to develop a platform to swap the variable chains of the immunoglobulin genes to change their specificity^11^. To date, there are no reports on the use of genomic editing in hybridoma cells to site-specifically modify antibodies with encodable linkers for bioconjugation purposes.

Here, we have developed a strategy to bypass these challenges in mAb modification via site-specific modification of the immunoglobulin gene within the hybridoma itself. Using CRISPR/Cas9 genomic editing, we have incorporated Sortase (LPETGG) and Flag (DYKDDDDK) tags at the C-terminal end of the CH3 heavy chain region in a mouse monoclonal antibody providing conjugation of cargoes without loss of antibody affinity, while ensuring optimal orientation of the antibody and minimizing steric hindrance or altered conformation of the complementarity-determining regions (CDRs). The strategy presented here reduces the time and cost it takes to make genetically encoded modifications to antibodies. This is a key development, as conjugatable antibodies have numerous applications in therapeutics and diagnostics.

## Results

### CRISPR-Cas9-mediated genomic editing of hybridoma cells

**Figure 1:**
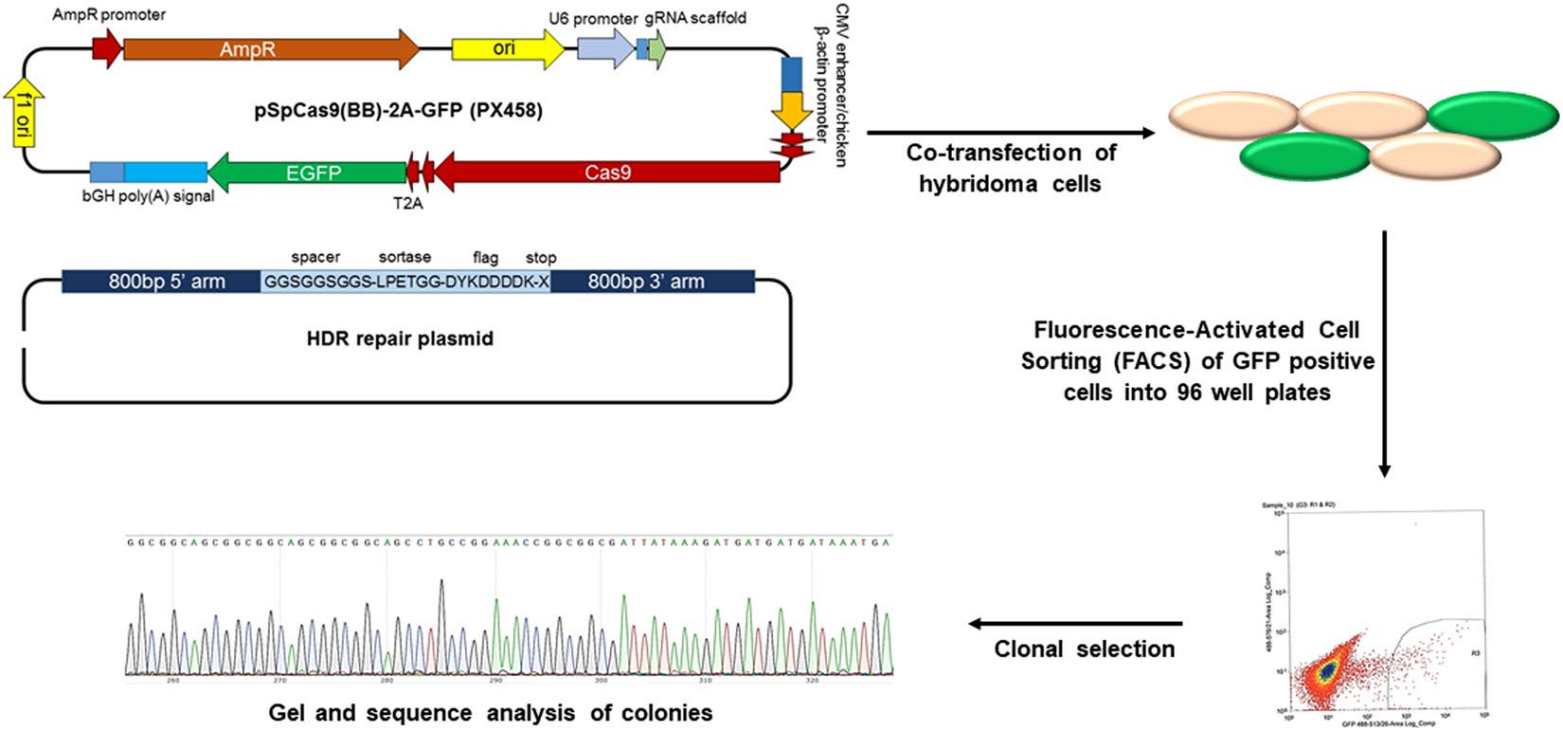
Illustration of CRISPR/Cas9 genome editing approach of hybridoma cells for site-specific modification of antibodies. Hybridoma cells were modified by co-transfection with plasmid expressing sgRNA and Cas9, and linearized HDR repair plasmid. After 48 hrs, GFP-positive cells were isolated using fluorescence-activated cell sorting (FACS) into 96 well plates and cultured. Clones were analyzed for incorporation if inserted tag by gel and sequence analysis.

**Figure 2:**
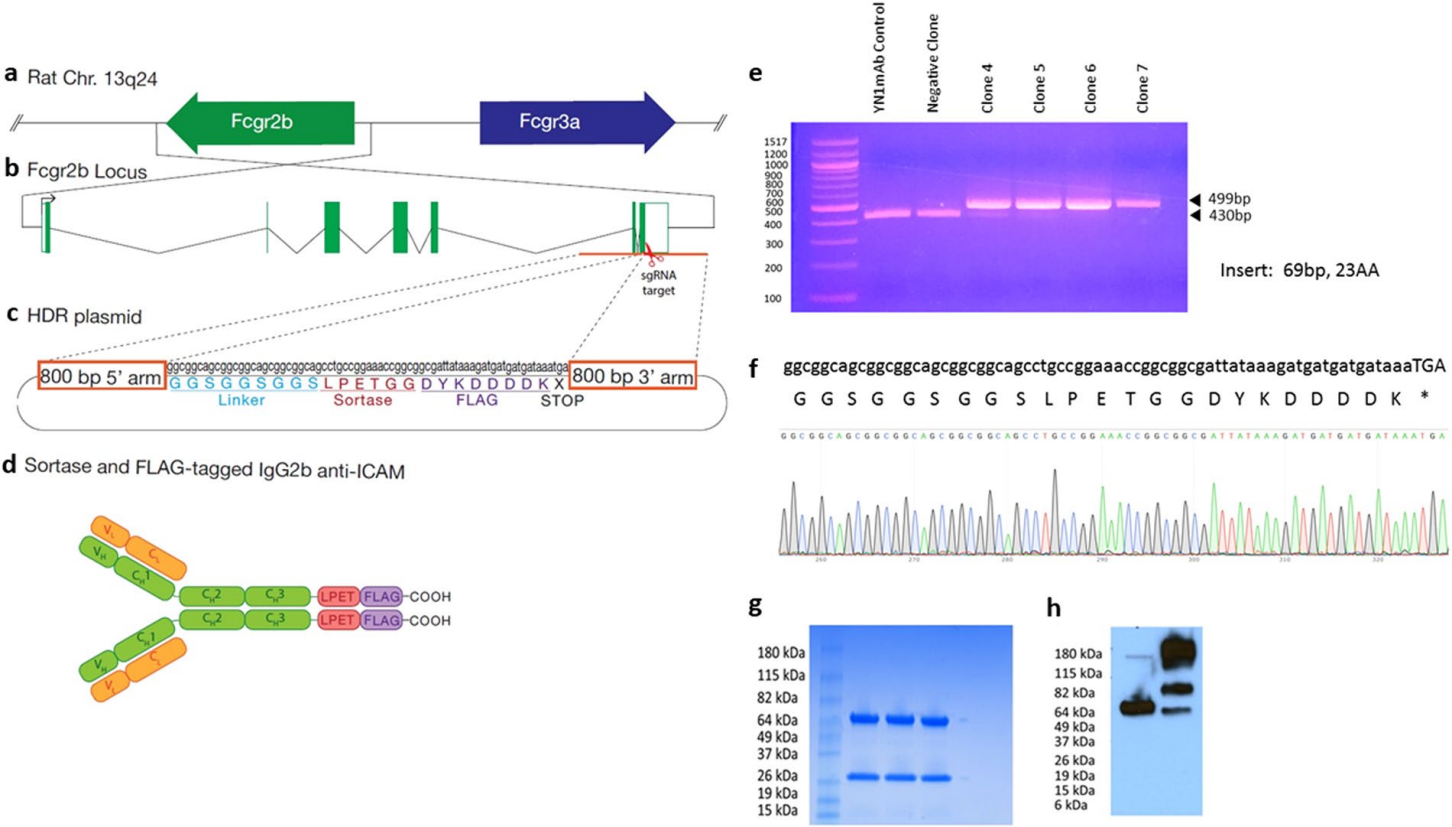
Schematic diagram and characterization of CRISPR/Cas9 genome editing of hybridoma cells for site-specific modification of antibodies. C-terminal end of Ig gamma-2B gene from (**a**) rat chromosome 13q24, (**b**) Fcgr2b locus, was modified by (**c**) homology directed repair to incorporate sortase and flag tags. sgRNAs with best On-Target/Off-Target scores were selected in the region near the IgG2b stop codon. (**d**) Anti-ICAMIgG2b antibodies were generated incorporating Sortase and FLAG tags at their C-terminal end. Confirmation of insert was performed by (**e**) Agarose gel analysis of PCR fragments from positive and negative clones. (**f**) Verification of integration in positive clones by sanger sequencing. (**g**) Coomassie blue stained gel of three positive clones. (**h**) Western blot of a positive clone under reducing and non-reducing conditions, stained with anti-Flag-HRP.

A rat hybridoma cell line that produces a mAb to mouse ICAM (anti-ICAM mAb) was used for site-specific modification. The sequence of the rat IgG2b constant region was identified in Ensembl and confirmed by sequencing. In order to genetically incorporate Sortase and Flag tags at the C-terminal end of antibody, two sgRNAs were selected in the region near the 3′ end of CH3 heavy chain stop codon. sgRNA 1 demonstrated greater efficacy and was the primary sgRNA used for generaetion of clones. We designed a donor construct containing two 800 bp homology arms flanking a 69 bp insert encoding a flexible leader sequence, Sortase, and FLAG tags (GGSGGSGGS-LPETGG-DYKDDDDK) to facilitate C-terminal modification of the Ig gamma-2B CH3 region using homology-directed repair (HDR). The sgRNAs were cloned into the pSpCas9(BB)-2A-GFP (PX458) plasmid which encodes the Cas9 nuclease as well as a GFP selection marker. Hybridoma cells were co-transfected with plasmid expressing both sgRNA and Cas9, and linearized HDR repair plasmid. After 48hrs, GFP-positive cells were isolated using fluorescence-activated cell sorting (FACS). Following co-transfection, the GFP-positive cells were clonally isolated by FACS into 96 well plates and cultured (Fig. 1). Genomic DNA was isolated for PCR analysis to identify the clones containing the inserted tags. Electrophoresis of the PCR products shows a 69 bp increase in product size with the incorporation of the 23 amino acid insert, whose sequence was confirmed by Sanger sequencing (Fig. 2e and f).

### Sortase-mediated site-specific conjugation of probes to mAbs modified using CRISPR-editing

**Figure 3:**
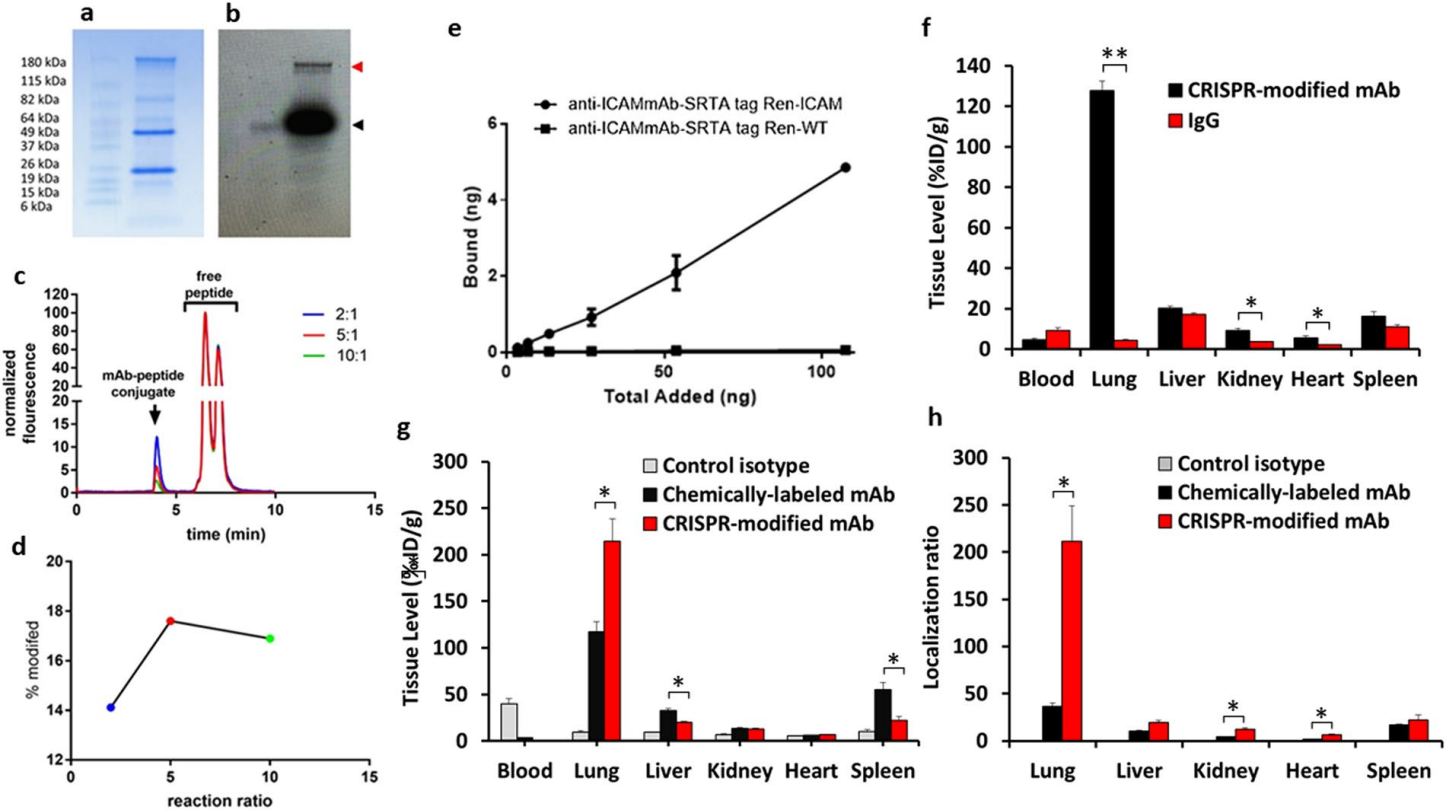
Conjugation efficiency and biodistribution analysis of CRISPR-modified antibody. (**a**) Coomassie blue stained gel of sortase-mediated conjugation of modified antibody to GGG-fluorophore. (**b**) Fluorescence scan of the antibody-fluorophore, stained with anti-Flag-HRP, showing the antibody conjugated fluorophore (red tab) compared to free fluorophore (black tab). (**c**) HPLC fluorescence trace antibody-fluorophore conjugates at different molar ratios. (**d**) Quantitative analysis of the percent of antibody modification. (**e**) Binding of ^125^I-labeled CRISPR-modified antibody to REN-ICAM and REN-WT cells. (**f**) Biodistribution of ^125^I-labeled CRISPR-modified anti-ICAM mAb in mice at 30 min. Tissue uptake is indicated as mean ± SEM (n = 3). Biodistribution analysis was performed comparing ^111^In-labeled site-specific CRISPR/Cas9- to chemically-modified mAb. (**g**) Biodistribution of ^111^In-labeled anti-ICAM mAb modified in mice at 30 min. Tissue uptake is indicated as mean ± SEM. (**h**) Localization ratio of selected organs. Significant differences were determined by t-test with Bonferroni correction to account for multiple comparisons.

SDS-PAGE analysis detected both light and heavy chains of antibody products of clones with inserts (Fig. 2g), while Western blot analysis with anti-Flag staining under reducing conditions shows only the heavy chain, indicating proper incorporation of the flag tag. Non-reducing conditions show higher molecular weight bands, indicative of heavy chain dimers and full-length antibody. Sortase-mediated conjugation efficiency of the modified anti-ICAM antibodies was evaluated using a Gly_3_-containing peptide that was labeled with Fluorescein isothiocyanate (FITC). Calcium-dependent enzymatic activity of Sortase A provided conjugation of a FITC-labeled Gly_3_-containing peptide GGGK-FITC to the CRISPR-modified anti-ICAM (Fig. 3a–d). Fluorescence scan (Fig. 3b) of the antibody site-specifically conjugated to GGG-FITC shows successful conjugation of the GGG-fluorophore (red tab) compared to free fluorophore (black tab). For an accurate assessment of fluorophore conjugation, an HPLC fluorescence trace of antibody-fluorophore conjugates was performed by sortase-mediated conjugation at different molar ratios of mAb to GGGK-FITC (Fig. 3c). The efficiency of the site-specific fluorescent labeling was quantified by HPLC with fluorescence detection. As shown in Fig. 3d, the percent antibody site-specifically modified were 14.1, 17.6, and 16.9 respectively for molar ratios of 1:2, 1:5, and 1:10. Although the conjugation efficiency is relatively modest, the cargo loading capacity may be enhanced by conjugation of multiple cargoes onto the GGG-peptide to form high payload peptide-drug conjugates capable of sortase-mediated conjugation. The issue of payload/carrier ratio is generally important for many targeted drug conjugates. Noteworthy, some cargoes including enzymes and other catalytic molecules such as nucleic acid-based therapeutics, as well as some imaging probes may provide the desirable effects at relatively low delivered dose. The effective dose and, accordingly, drug load conjugated to ligands likely be different for given pharmacological intervention, and, therefore, we believe that this issue should be addressed specifically for each agent, in course of envisioned industrial and clinical translation of our methodology.

### Targeting features of site-specifically modified antibody *in vitro* and in *vivo*

CRISPR-modified ^125^I-labeled anti-ICAM selectively bound to ICAM-expressing, but not to negative control cells *in vitro* (Fig. 3e) and accumulated in the lungs of mice after intravenous injection (Fig. 3f), exceeding the uptake of isotype matched control IgG by more than order of magnitude (126.7 ± 4.3 vs. 10.4 ± 1.2%ID/g for CRISPR anti-ICAM mAb vs. IgG, respectively), matching previously reported pulmonary vascular immunotargeting of ^125^I-labeled anti-ICAM^12–16^. A biodistribution study (Fig. 3g and h) was carried out to investigate differences in organ biodistribution between anti-mICAM mAbs that were radiolabeled by non-specific-chemical conjugation vs. site-specific conjugation. Chemical conjugation was carried out by conjugating the native anti-ICAM mAb with S-2-(4-Isothiocyanatobenzyl)-1,4,7,10-tetraazacyclododecane tetraacetic acid (DOTA-SCN), an amine-reactive isothiocyante derivative of DOTA radiometal chelator, followed by one-step labeling using established methodologies^17^. Quantification of the incorporated DOTA by a lead-arsenazo assay^18^ demonstrated 2–3 chelating groups per antibody. For site-specific radiolabeling of the antibody, a two-step labeling procedure was used. GGGK-DOTA peptide was first labeled with ^111^In isotope under metal free conditions with radiometal incorporation into the peptide tracked using instant thin layer chromatography (TLC), which demonstrated near-complete (>95%) chelation. The GGGK-DOTA-^111^In was then conjugated to modified antibody in the presence of sortase A enzyme. The immunoreactivity of the antibodies modified by both non-specific chemical conjugation and site-specific modification was >80% by a modified Lindmo method^19^. Comparing with anti-ICAM labeled with ^111^Indium using standard non-specific chemical conjugation of the chelating group DOTA, and anti-ICAM labeled with ^111^In via DOTA conjugated using sortase A in a site-specific fashion to the sequence introduced by CRISPR-editing, there was markedly reduced uptake by clearing organs including liver and spleen, accompanied with nearly 100% improvement of the pulmonary immunotargeting (214 ± 23.97%ID/g compared to 117.49 ± 10.58%ID/g respectively). As result, lung to blood ratio of %ID/g increased more than five-fold for site-specific vs conventionally labeled anti-ICAM mAb (211.08 ± 37.61 vs 36.29 ± 3.87). These marked differences in biodistribution demonstrate the profound effect site-specific conjugation can have on reducing non-specific uptake from the major reticuloendothelial system organs the liver and spleen, while significantly enhancing target-specific uptake.

## Discussion

We describe a CRISPR/Cas9 genomic editing strategy for development of antibodies in hybridoma cells ready for site-specific conjugation. An anti-mICAM monoclonal antibody hybridoma cell line was genetically modified at the C-terminal end of the immunoglobulin CH3 heavy chain constant region to incorporate a sortase tag (for conjugation) and a FLAG tag (for characterization and affinity purification). The CRISPR/Cas9 editing technique allows for easy, rapid and efficient engineering of antibodies in the hybridoma cells themselves, without the need to sequence antibody variable regions and clone them into producer cells lines such as CHO cells. This eliminates several expensive and time-consuming steps in the production of site-specifically conjugated antibodies.

The current antibody-drug conjugates on the market use chemical conjugation to lysine or cysteines on the antibody^4,20,21^. In conventional chemical conjugation, there is limited control over the location and extent of chemical conjugation. Over-modification of the antibody can lead to aggregation, loss of stability and functionality, and decreased circulation time or increased clearance by the reticuloendothelial system. Site-specific conjugation promises to maintain homogeneity and ensure reproducible manufacture of antibody conjugates^3–5^. Genetic incorporation of additional amino acids and sequences into antibodies has been used to develop more effective bioconjugate therapeutics through site-specific conjugation. Innovative antibody variants have been engineered by incorporation of cysteines at different sites of the IgG for drug conjugation, called THIOMAB-drug conjugates (TDC), which have demonstrated improved homogenous site-specific antibody-drug conjugate production^22,23^. Another strategy has been incorporation of unnatural amino acids such as with para-acetylphenylalanine (pAcPhe) and para-azidomethyl-l-phenylalanine (pAMF) via aminoacyl-tRNA synthetases that recognize the UAAs and incorporate them into amber stop codons inserted at desired sites^24–27^. Enzyme-assisted ligation such as by formylglycine-generating enzyme (FGE)^28^, bacterial transglutaminase (BTG)^29,30^, and bacterial enzyme sortase A (SrtA)^31^ can be used for site-specific conjugation to genetically incorporated linkers. The bacterial-derived transpeptidase enzyme sortase catalyzes conjugation between proteins tagged with LPXTG motif and triglycine containing moieties^32–34^. Recently, Pan L. *et al*. generated potent antibody-drug conjugates using sortase-mediated conjugation of sortase tagged antibodies to GGG-modified antineoplastic agents. The antibodies were developed by cloning the antibody heavy and light chain variable regions into the pFUSE-CHIg-hG1 and pFUSE2-CLIg-hK plasmids. The pentapeptide sortase tag LPETG was incorporated into the C-terminals of the heavy or light chains. The plasmids were then transiently co-transfected into CHO cells and scaled up for modified antibody production. This technique differs from our current strategy in that it is transient transfection and it requires variable chain sequencing and cloning. However, this study showed the benefits of site-specific modification of antibodies for bioconjugation and demonstrated enhanced control of drug-antibody ratio. Site-specific modification facilitated lower drug-antibody ratios, which are necessary to improve pharmacokinetic and homogeneity to reduce drug-related toxicity^35^. The conjugation tag size and its effect on the structural stability of the carrier protein can influence its immunogenicity. Larger tags are typically more likely to alter the structural features of the carrier protein leading to exposure of hidden hydrophobic amino acids leading to enhancement of immunogenicity. The sortase-recognition motif LPETG immunogenicity is expected to be minimal, if any, in view of its tiny size. Incorporation of larger tags or the use of non-specific chemical conjugation reactions in most cases should have greater detrimental effects on antibodies than compared to site-specific approaches, resulting in poor biodistribution and pharmacokinetics. Furthermore, many potential applications envisioned for the vascular immunotargeting will take place in acute settings, when single injection of the conjugate may provide the decisive effect without posing danger of immune reactions.

The CRISPR/Cas9 system is an efficient and cost-effective genomic engineering platform that could be very beneficial in the field of antibody engineering. Various primary cells and cell lines have been genetically reprogrammed using CRISPR/Cas9 to knockout or knockin specific genes^36–43^. In one study, a multiplexed CRISPR/Cas9 strategy was used to generate a triple knockout CHO cell line^44^. The genes BAK, BAX, and FUT8 were simultaneously disrupted in CHO cells to provide resistance to apoptosis and enhanced survival, as well as defucosylated proteins. These modifications can enhance antibody yields and antibody dependent cellular cytotoxicity (ADCC) for use in cancer immunotherapeutics^45^. Such genetic engineering strategies for cellular reprogramming can be of great benefit to industry. One downside of these approaches is that they ultimately require sequencing and cloning in of the antibody variable chains.

**Figure 4:**
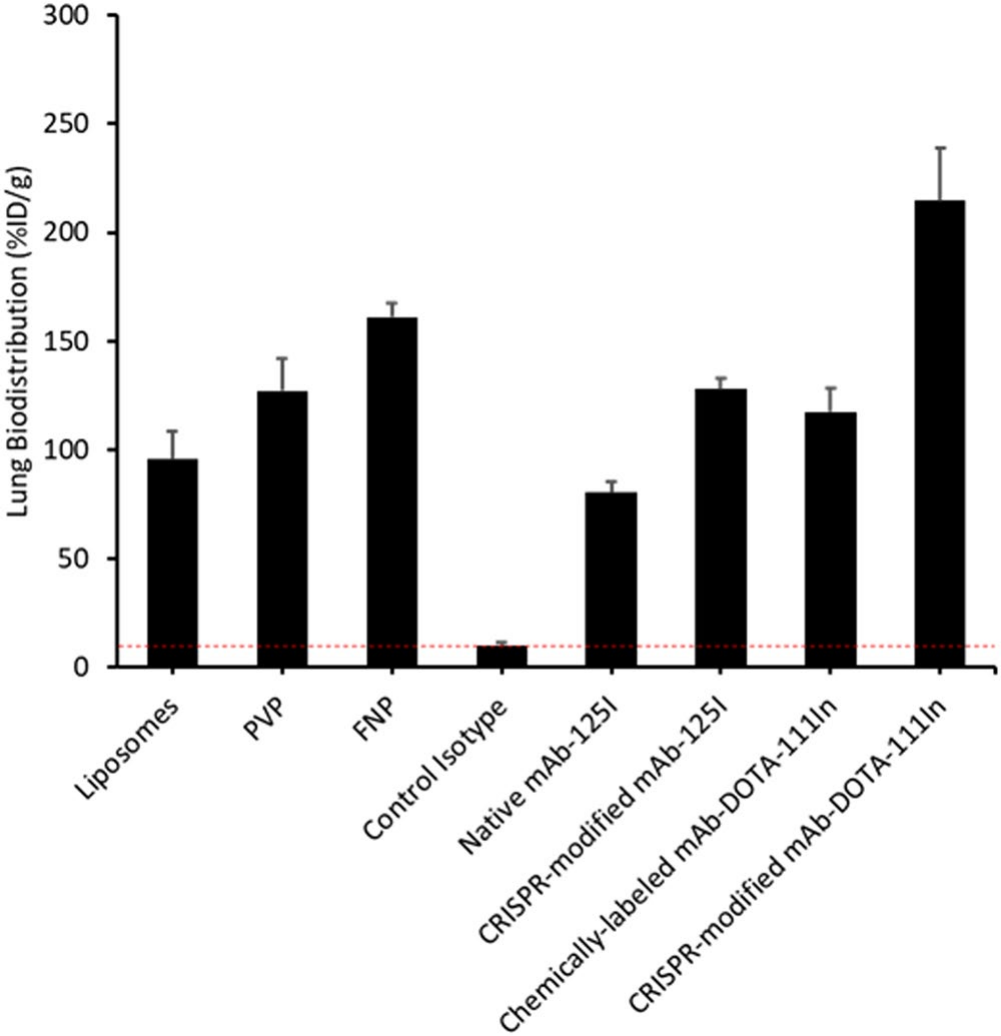
Comparison of the pulmonary targeting of various ICAM-1 directed agents. Lung Biodistribution, calculated as percent injected dose per gram (%ID/g) of tissue. Control Isotype – purified mouse IgG2b antibody, PVP - 100-nm polyvinylphenol particles, liposomes - 150-nm PEGylated immunoliposomes, and FNP - ferritin nanoparticles^15,46–48^.

Here we utilized a CRISPR/Cas9 system to site-specifically modify anti-mICAM antibody hybridoma cell line to generate modified antibodies equipped with a sortase tag for site-specific bioconjugation of a range of moieties. The modified anti-ICAM antibody was used to target the pulmonary endothelium which plays a pivotal role in pathological conditions such as acute respiratory distress syndrome, pulmonary arterial hypertension, pulmonary embolism, and others. Vascular immunotargeting of the pulmonary endothelium can be very valuable in targeted drug delivery for treatment of pulmonary diseases. The CRISPR-modified anti-ICAM antibody demonstrated successful site-specific conjugation using a GGGK-peptide modified with both fluorophores or radioisotopes, without any interference in the functional activity of the antibody. Analysis of the biodistribution study comparing the conventional chemically conjugated DOTA-^111^In to genetically modified site-specifically conjugated GGG-DOTA-^111^In, demonstrated a significant increase in lung targeting with %ID/g of 214 ± 23.97 and localization ratio of 211 ± 37.6, which is remarkable when compared to non-modified antibody and multivalent nanoparticle-based targeting strategies. CRISPR-modified antibody demonstrated lung targeting superiority over reported ICAM-targeted nanoparticles such as liposomes, polyvinylphenol particles, and ferritin nanoparticles (Fig. 4)^15,46–48^. A marked decrease in non-specific uptake by liver and spleen is also observed by the CRISPR-modified antibody. The detrimental effects of random chemical conjugation and non-specific ^125^I-based radiolabeling could explain the poor lung targeting and increased uptake by liver and spleen. Site-specifically radiolabeled antibody through enzyme-mediated conjugation of DOTA-^111^In provided superior targeting over ^125^I-based labeling strategy, and is found to be advantageous approach for both conjugation and radiolabeling. The large effect size of the particular radiolabeling approach is observed by comparison of localization ratio for CRISPR-modified mAb-DOTA-^111^In at 211 ± 37.6, to non-specifically iodinated CRISPR-modified mAb at 43.7 ± 5.9 and iodinated native mAb at 23.4 ± 2.7. Moreover, through the CRISPR-based modification, no change was made to the Fc region, as compared to conventional conjugation methods which could lead to perturbation of the Fc domain or over-modification of the antibody. These exciting results emphasize the importance of site-specific antibody labeling in functionality and targeting efficiency, and have outlined a cost-efficient technique to generate site-specifically conjugated antibodies in hybridoma cell lines.

By dramatically reducing the cost, labor, and time required to make genetically encoded modifications to antibodies produced by hybridoma cells, this innovation has the potential to allow academic labs and small drug companies to experiment with and tweak candidate antibody-based biologic therapies, something that has heretofore mostly taken place within large pharmaceutical companies. Our genetic modification of hybridoma cells allows for the rapid development of antibodies that are primed for site-specific conjugation. By modifying the antibody with encodable linkers primed for site-specific modification in the hybridoma cells, we bypass the challenges associated with having to sequence variable chains and subsequent modification and cloning into stable production cell line. Finally, the site-specific encodable linkers could provide greater control over the number of drug molecules conjugated as well as enhance the reproducibility and homogeneity of the antibody-drug conjugates.

## Methods

### Cell culture

Rat hybridoma YN1/1.7.4 (ATCC CRL-1878) cell line was grown in RPMI 1640 medium supplemented with 10% fetal bovine serum supplemented with, 2 mM L-glutamine, 100 U/ml penicillin, and 100 μg/ml streptomycin (Life Technologies, Carlsbad, CA). REN-wild type (human mesothelioma) and REN-mICAM cells were grown to confluence in RPMI 1640 medium with 10% fetal bovine serum supplemented with 2 mM L-glutamine, 100 U/ml penicillin, and 100 μg/ml streptomycin (Life Technologies, Carlsbad, CA).

### sgRNA target selection

YN1/1.7.4 rat hybridoma cell line expressing anti-mICAM monoclonal antibody (isotype IgG2b) was used for genomic editing. Rat IgG2b constant region sequence was identified from Ensembl rate genome build Rnor_6.0 using transcript Igh-6–201 ENSRNOT00000045874, and confirmed by sanger sequencing. For CRISPR modification of the C-terminal end of the antibody, the region near the stop codon at the 3′ end of the exon encoding the C-terminus of the CH3 heavy chain was chosen for sgRNA selection. Benchling CRISPR sgRNA design tool was used to select two sgRNAs, one upstream and one downstream of the stop codon. sgRNA 1 (agaaagctctcaggtcctaa) located 27 bp downstream of the end of stop codon had an On-Target/Off-Target score of 80.6/38.1. sgRNA2 (gggtctgcacaatcaccacg) located 38 bp upstream of the end of stop codon had an On-Target/Off-Target score of 85.7/45.3. Both sgRNAs were tested for the C-terminal modification of the antibody. sgRNA 1 was more efficacious and was the principle sgRNA used to produce the clones.

### Construction of the sgRNA-expression plasmid

For construction of the sgRNA-expression plasmids, a technique similar to the Zhang protocol^7^ was used. pSpCas9(BB)-2A-GFP (PX458) plasmid (addgene # 48138) was used for scarless cloning in of the sgRNA. Two guanines were additionally inserted into the 5′ end of the sgRNA to enhance the U6 transcription. The + /− strands of the sgRNA were flanked with overhangs CACC and CAAA respectively, for ligation into BbsI site in the pSpCas9 plasmid. DNA oligos were purchased from IDT (Integrated DNA Technologies, Coralville, Iowa) and resuspended to concentration of 100uM. The + /− strands oligos were phosphorylated with T4 PNK enzyme and annealed in a thermocycler with the following conditions: 30 min at 37 C; 5 min at 95 C; and 5 min at 25 C. The oligos were diluted 1:200, and cloned into the pSpCas9(BB)-2A-GFP (PX458) plasmid. Digestion and ligation was carried out using FastDigest BbsI restriction enzyme and T7 ligase for 1 h with 6 cycles of 37 C for 5 min; 21 C for 5 min. The ligation reaction was transformed into One Shot Top10 competent e. coli (Thermo Fisher Scientific, Waltham, MA) using manufacturer transformation protocol. sgRNA insertion was confirmed using sanger sequencing.

### HDR repair plasmid construction and hybridoma transfection

800 bp complementary sequence upstream and downstream of the stop codon was used to design the left and right homology arms. The insert GGSGGSGGS-LPETGG-DYKDDDDK was placed in between the homology arms. PAM sequences of the sgRNAs in the HDR were mutated to prevent re-cutting by the Cas9. The HDR sequence was flanked by BamHI and HindIII restriction enzyme sites for insertion into the pUC19 vector. A gBlock with this sequence was purchased from IDT (Integrated DNA Technologies, Coralville, Iowa) and cloned into TOPO vector using Zero Blunt TOPO PCR cloning kit (Thermo Fisher Scientific, Waltham, MA). The HDR-TOPO plasmid was digested using BamHI and HindIII, and run on a 1% (wt/vol) agarose gel. The digested HDR was ligated into pUC19 vector. pUC19-HDR plasmid was then linearized near the 5′ end of the upstream homology arm of the HDR using EcoRI enzyme. The cas9 plasmid and the linearized HDR plasmid were co-transfected into YN1 hybridoma cells with GeneJammer transfection reagent (Agilent Technologies, Santa Clara, CA) using manufacturer’s protocol. After 48 hours the transfection efficiency was evaluated, followed by clonal cell isolation of GFP expressing cells.

### Clonal cell isolation

BD AriaII FACS cell sorter was used for clonal cell isolation of the GFP positive cells. A minimum of 1 million cells was placed in a maximum of 500 μL RPMI media with 2% serum. The cells were run through a 70 μm cell strainer. Single cells were deposited in 96 well plate, and brought up to 200 μL with serum rich RPMI media. After 5–7 days the cell dense wells were scaled up into 24 well then 6 well plates, at which time genomic extraction and Sanger sequencing was carried out.

### Genomic DNA analysis

The YN1mAb hybridoma genomic DNA was extracted using GeneJet genomic DNA purification kit from Thermo Fisher Scientific (Waltham, MA). PCR analysis was carried out using primers covering the C-terminal end of the YN1mAb. Herculase II Fusion DNA Polymerase Kit from Agilent Technologies (Santa Clara, CA) was used for PCR analysis. The PCR amplicons were run on a 2% (wt/vol) agarose gel and stained with ethidium bromide. Sanger sequencing was carried out for confirmation of the insert.

### Western blot analysis

The tagged YN1 mAb clones were analyzed by western blot using anti-Flag staining. Samples were run under reducing and non-reducing conditions on sodium dodecyl sulfate polyacrylamide gel electrophoresis (SDS-PAGE), 4–15% gradient gel (Mini-PROTEAN® TGX™ Gel, Bio-Rad, Hercules, CA). Gels were transferred to PVDF membrane (Millipore, Billerica, MA). Membrane was blocked for 1 h with 3% non-fat dry milk in TBS-T (100 mM Tris, pH 7.5; 150 mM NaCl; 0.1% Tween 20). The membrane was incubated with anti-FLAG M2 antibody (F3165; Sigma-Aldrich, St. Louis, MO), followed by goat anti-mouse HRP secondary antibody (ab6789; Abcam, Cambridge, MA). For chemiluminescence western blot analysis of the modified antibodies, Amersham ECL Western Blotting Detection Reagent (GE Healthcare Bio-Sciences, Pittsburgh, PA) was used.

### Sortase-mediated conjugation

The antibody (500 nM) and GGGK-FITC peptide (Thermo Fisher Scientific, Waltham, MA) were incubated at different molar ratios in presence of 10 μM sortase A enzyme and 1 mM Calcium in Tris-buffered saline (TBS) buffer for overnight at rt. For quantification of the number of fluorophores sortase-conjugated to antibody, size-exclusion high-performance liquid chromatography (SEC-HPLC) was performed using a BioSep SEC-s3000 column (Phenomenex, Torrance, CA). The samples were run by isocratic method with 100% PBS pH 7.4.

### Antibody 125-I radiolabeling and cell binding assay

The tagged YN1 mAb was radiolabeled with Na^125^I using Pierce Iodination Beads. The reaction was carried out for 15 min at ambient temperature. Free ^125^I was removed by Quick Spin Protein Columns (G-25 Sephadex, Roche Applied Science, Indianapolis, IN). REN wild type and REN-mICAM cells were plated on 24 well plates (Corning Inc., Corning, NY) and grown to confluence (10^5^ cells/cm^2^). The radiolabeled antibodies were added to the corresponding cells and incubated for 1 hour at ambient temperature. After 1 hour, the cells were washed three times with ice-cold Hank’s Balanced Salt Solution (HBSS, Corning Cellgro, Manassas, VA), and then lysed with lysis buffer (1% Triton X-100, 1 M NaOH). The lysates were measured using Wallac 1470 Wizard™ gamma counter (Gaithersburg, MD). The bound radioactive antibodies were plotted against the corresponding total amount added.

### ^111^In labeling of antibody using site-specific enzymatic labeling

^111^InCl_3_ was purchased from Nuclear Diagnostic Products (Cherry Hill, NJ). 700 μCi of the isotope (in approximately 50 μL of 0.05 M HCl) as provided by the supplier was diluted into 50 μL of metal-free 0.5 M tetramethyl ammonium acetate, pH 4.5. Buffers were rendered metal-free by treatment with Chelex 100 resin (Bio-Rad Laboratories, Hercules, CA). In a typical labeling reaction, 2 μL of GGGK-DOTA peptide (Thermo Fisher Scientific, Waltham, MA) in metal free water was added to 70 μL of the 111-indium solution and the pH adjusted to 4.5. This mixture was incubated at 37 °C for 1 hour and radiometal incorporation was confirmed by instant thin layer chromatography in a mobile phase of 0.9%NaCl/10 mM NaOH which demonstrated 97% incorporation of isotope into the DOTA-modified peptide. The labeled peptide was added to 500 μL of YN1-LPET (at 0.5 mg/mL) with the addition of 1 M Tris pH 8 until the pH reached 7–7.5. Recombinant sortase A was then added at a final concentration of 30 μM and CaCl_2_ added at a concentration of 1 mM. The sortase conjugation was performed overnight at room temperature. The following day a 10-DG desalting column was equilibrated with PBS and the reaction mixture purified into 2 mL final volume. The purified mixture was used as the injection formulation in biodistribution studies without further modification. Instant thin layer chromatography in mobile phases of 0.9%NaCl/10 mM NaOH and 10 mM EDTA demonstrated >99% radiochemical purity (specific activity of 0.04 Ci/g) of the enzymatically labeled antibody. Based on a reaction ratio of 5 peptides per YN1-LPET, the reaction yield was approximately 7%.

### ^111^In labeling of antibody using bifunctional chelators

Native YN1 monoclonal antibody was rendered metal-free by treatment with 5 mM DTPA followed by buffer exchange into metal-free bicarbonate buffer at pH 8.5 using a 10-DG desalting column (Bio-Rad Laboratories, Hercules, CA). In a typical reaction, 1.2 mg of this antibody was reacted with 275 μg of DOTA-SCN at pH 8.5 for 1 hour at ambient temperature followed by purification into metal-free 50 mM ammonium acetate, 150 mM NaCl, pH 4.5 using a 10-DG desalting column previously rendered metal-free by treatment with DTPA. The resultant metal-free mAb-DOTA conjugate was labeled with ^111^InCl_3_ as described above, with purification into PBS. Typical labeling reactions using this approach resulted in >99% radiochemical purity by instant thin layer chromatography.

### Biodistribution of antibodies *in vivo*

Animal experiments were carried out according to protocol approved by Institutional Animal Care and Use Committee (IACUC) of the University of Pennsylvania. Radiolabeled antibodies were injected IV via retro-orbital injection in C57BL/6 J mice (The Jackson Laboratory, Bar Harbor, ME). After 30 min, blood was collected and the organs were harvested. Radioactivity of the samples was determined with Wallac 1470 Wizard™ gamma counter. The weight of the organs and their radioactivity compared to a standard of the injection formulation were used to calculate percent injected dose per gram (%ID/g).

### Statistical analysis

Statistical analysis was performed using student t-test with Bonferroni correction. Differences were considered statistically significant at p < 0.05.
